# The effect of different endoscopic tattooing doses in laparoscopic colorectal tumor surgery

**DOI:** 10.1097/MD.0000000000050870

**Published:** 2026-09-25

**Authors:** Ali İmran Küçük, Onur Sakalli, Bülent Toksöz, Mehmet Aziret

**Affiliations:** a Department of Surgical Oncology, Balikesir Atatürk City Hospital, University of Health Sciences, Balikesir, Türkiye; b Department of General Surgery, Balikesir Atatürk City Hospital, University of Health Sciences, Balikesir, Türkiye; c Department of Gastrointestinal Surgery, Balikesir Atatürk City Hospital, University of Health Sciences, Balikesir, Türkiye.

**Keywords:** colorectal neoplasms, laparoscopy, tattooing

## Abstract

We aimed to demonstrate the intraoperative effectiveness of different doses of preoperative endoscopic tattooing in patients undergoing laparoscopic surgical treatment for colorectal cancer or tumors. According to the tattooing dose administered before laparoscopic surgery, patients were divided into 3 groups: Group 1, tattooing dose ≥ 1.0 cc (n = 35); Group 2, 0.5–<1.0 cc (n = 33); Group 3, <0.5 cc (n = 48). The groups were statistically compared in terms of clinical characteristics, surgical parameters, histopathological features, and ease of identification. A total of 116 patients were included in the study; the mean age was 63.8 (10.5) years, and most were male (68.1%). The rate of easy intraoperative identification during laparoscopic colorectal surgery was 88.7% (103/116). There were no statistically significant differences between the groups in terms of age, sex, body mass index, anesthesia score, tumor localization, operative time, tumor diameter, malignancy rate, neoadjuvant therapy, TNM and T stage, or number of resected lymph nodes (*P* > .05). Additionally, there were no statistically significant differences among the 3 groups regarding ease of identification and spillage of the dye into the peritoneum (*P* = .706 and *P* = .207, respectively). In laparoscopic colorectal tumor surgery, low-dose endoscopic tattooing provides similar outcomes in terms of ease of identification compared to high-dose tattooing. Low-dose tattooing is associated with lower cost and reduced risk of spillage into the peritoneum.

## 1. Introduction

In the treatment of colorectal cancer (CRC) or tumors, preoperative endoscopic tattooing has been applied in recent decades. With the spread of minimally invasive methods, especially laparoscopic and robotic surgery, new challenges regarding tumor localization have emerged. The most important of these is the accurate identification of the tumor site during surgery.^[1]^ In MIM, the absence of tactile sensation, atypical or non-visible tumor localization, and particularly small-sized colonic lesions make it difficult to determine the exact location of the mass.^[2,3]^

Although the location of lesions is defined according to the segment of the colon in preoperative colonoscopy or rectoscopy, this can be limited due to anatomical variations. Misidentification or incomplete identification of tumor localization may result in resection of tumor-free segments, failure to remove the tumor in accordance with oncological principles, conversion to open surgery, or the need for additional resections.^[4,5]^

For these reasons, preoperative endoscopic tattooing is performed to facilitate tumor identification before surgery. Over the years, various methods have been used for marking, but studies have shown that the most effective method is the use of endoscopic dyes.^[6–8]^ Since India ink must be diluted before each use and may cause tissue inflammation, Spot®, derived from purified carbon particles and refined India ink, has been introduced. The use of this dye has shown no complications such as pain, fever, or abscess formation in patients, while its effectiveness remains high.^[9]^

Significant differences exist regarding the timing and techniques of endoscopic tattooing.^[10]^ In our study, we investigated the effectiveness of different tattooing doses in terms of ease of identification during laparoscopic surgery.

## 2. Materials and methods

### 2.1. Patients and ethics

The study was conducted between November 2018 and April 2025 in the Departments of General Surgery, Balikesir Atatürk City Hospital and Sakarya Training and Research Hospital. Ethical approval was obtained from the Balikesir Atatürk City Hospital Scientific Research Ethics Committee (Decision No. 2025/10/118). Patients who underwent laparoscopic surgery with a diagnosis of CRC were included. All patients were retrospectively evaluated for age, sex, body mass index (BMI), American Society of Anesthesiologists score, tumor localization, operative time, the largest tumor diameter excised, malignancy rate, neoadjuvant therapy, tumor-node-metastasis (TNM) stage, number of resected lymph nodes, T stage, and ease of identification. The diagnosis of CRC was established by preoperative colonoscopic or rectoscopic biopsy. Clinical staging was performed using contrast-enhanced computed tomography and pelvic magnetic resonance imaging. Positron emission tomography/computed tomography was performed in suspicious cases. Ethical committee approval was obtained.

### 2.2. Methodology

A total of 116 patients who underwent preoperative colonoscopic or rectoscopic submucosal tattooing and laparoscopic surgical treatment were included. Patients were retrospectively evaluated and divided into 3 groups:

- Group 1: tattooing dose ≥ 1.0 cc (n = 35)
- Group 2: 0.5 –<1.0 cc (n = 33)
- Group 3: tattooing dose < 0.5 cc (n = 48)

Patients were statistically compared in terms of clinical and histopathological values.

### 2.3. Endoscopic tattooing

Tattooing was performed within 1 to 3 days before surgery by the same physician. For rectum or sigmoid lesions, rectosigmoidoscopy without anesthesia was used. For proximal descending colon lesions, anesthesia was administered outside the operating room. Tattooing was performed 1 to 2 cm distal to the tumor, after submucosal inflation with 1 cc saline. Doses of  ≥ 1.0 cc, 0.5–<1.0 cc, or < 0.5 cc of dye were injected according to the group. At least one-third of the lumen was stained. Spot® (GI Supply, Camp Hill), a carbon-based dye with minimal allergic risk, was used (Figure 1).

**Figure 1.**
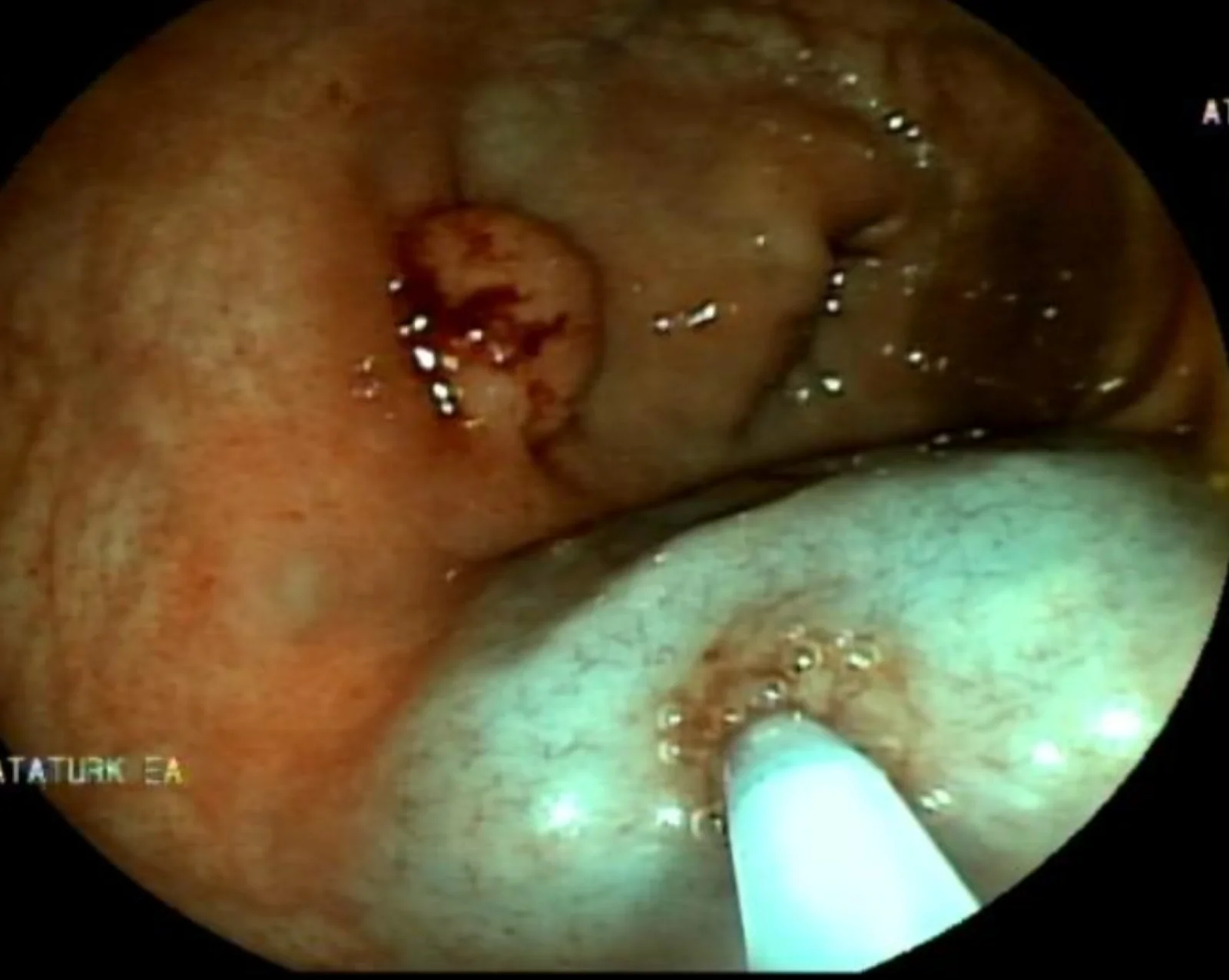
Preoperative endoscopic tattooing with Spot® dye performed 1 to 2 cm distal to the tumor lesion.

### 2.4. Inclusion and exclusion criteria

The inclusion criteria were patients aged 18 years or older and patients diagnosed with CRC or tumors by biopsy. Patients who were scheduled for laparoscopic surgery and those who underwent preoperative endoscopic tattooing were also included. In addition, patients whose tumors or polyps could not be endoscopically resected were eligible for the study.

The exclusion criteria included patients who underwent open colorectal surgery, those without a diagnosis of CRC or tumor, and patients younger than 18 years.

### 2.5. Laparoscopic surgery

After general anesthesia, patients were positioned according to tumor localization. Antibiotic prophylaxis was administered in all cases. Laparoscopic colorectal surgery was performed by the same surgeon using the North American technique. Depending on tumor location, laparoscopic low anterior resection, anterior resection, left hemicolectomy, or right hemicolectomy was performed. Tattoo identification was routinely evaluated (Fig. 2–4) During the laparoscopic entry, peritoneal spillage of the dye was systematically assessed. Spillage was identified via direct visual inspection by the surgical team immediately upon entering the peritoneal cavity, defined as the presence of free carbon dye/stain on the adjacent visceral peritoneum or pelvic floor, and was documented in the standard operative reports.

**Figure 2.**
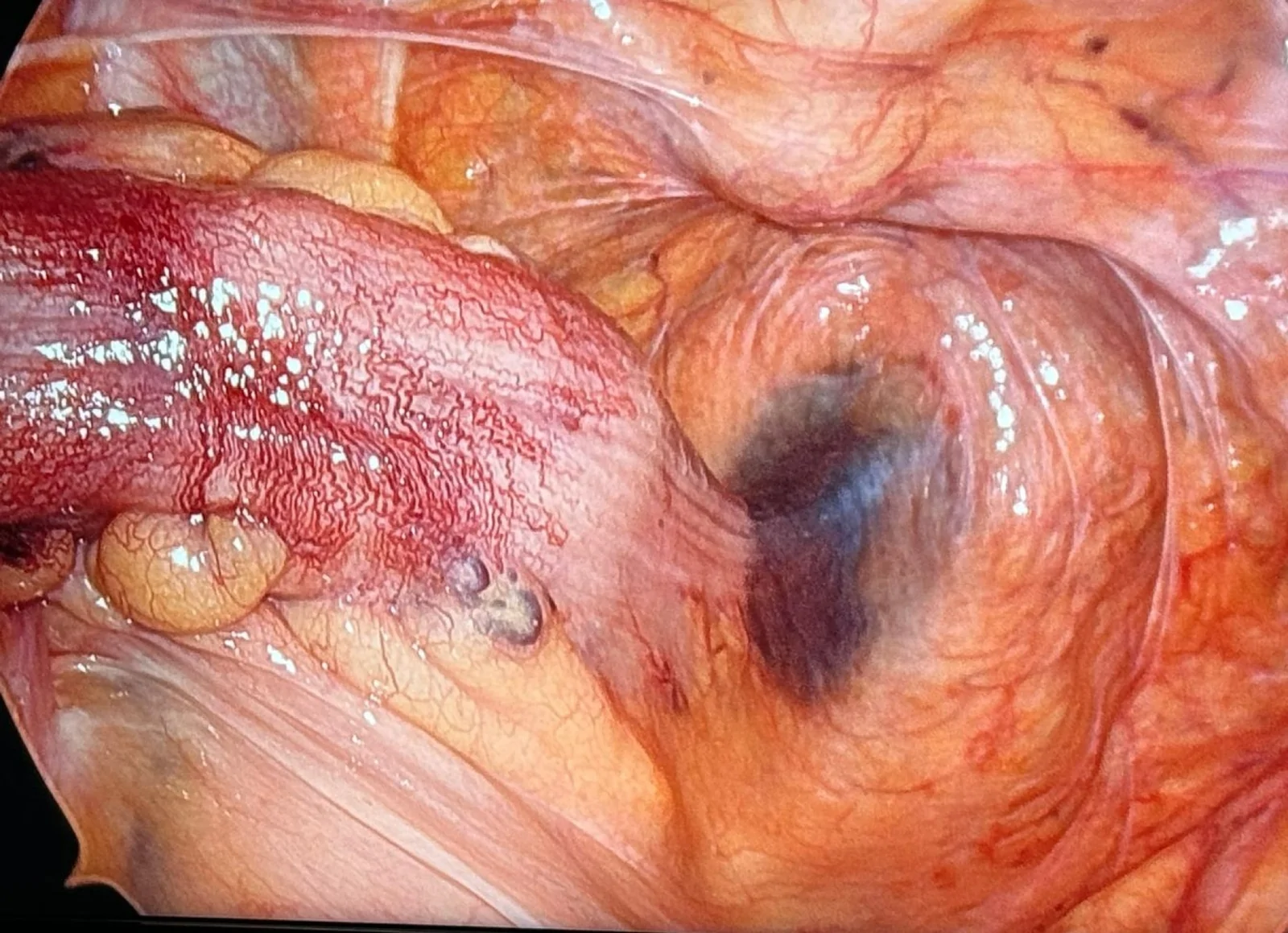
Intraoperative laparoscopic view showing the direct visualization of the tattooed segment during dissection.

**Figure 3.**
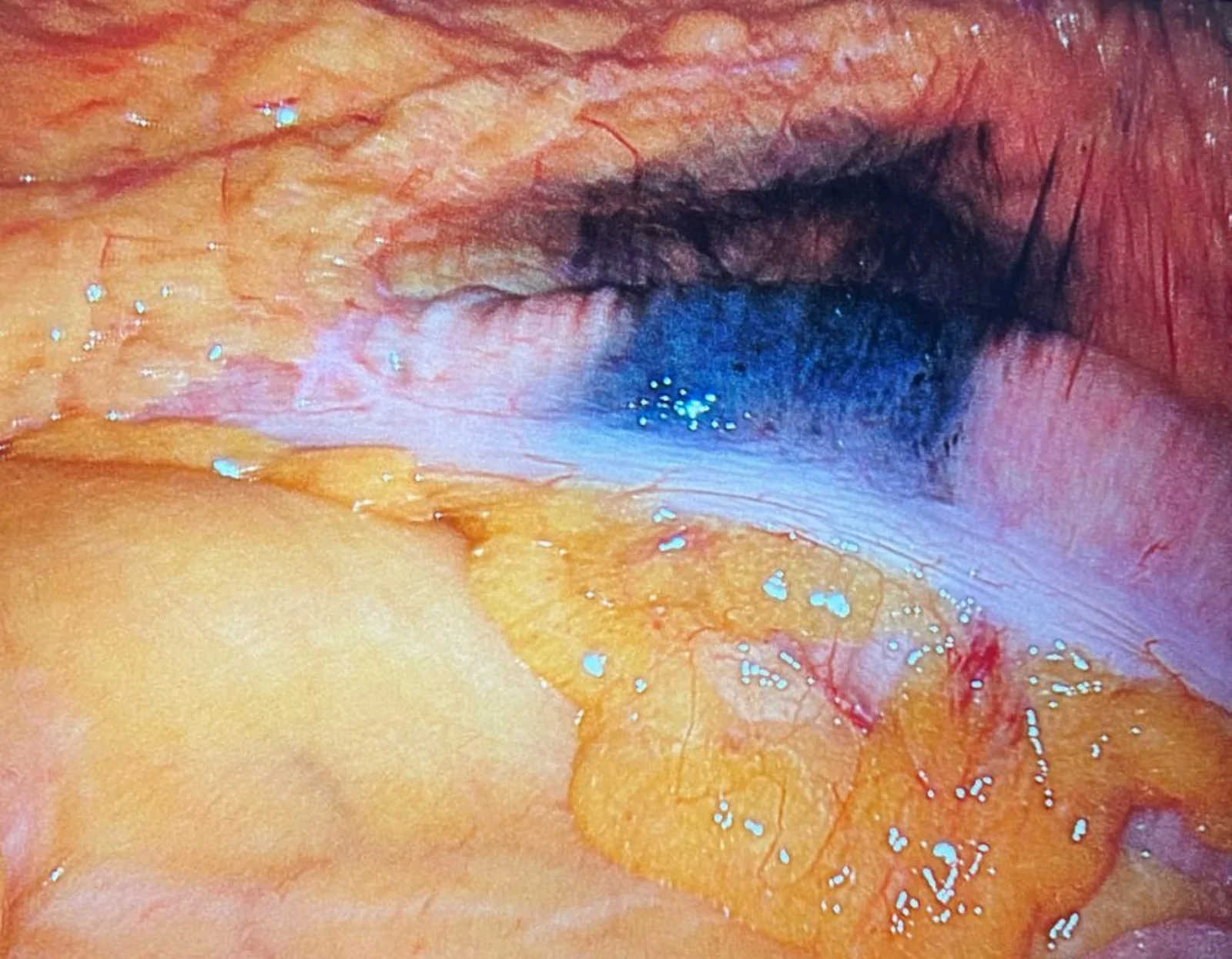
Intraoperative laparoscopic identification of the surgical margin marked by the reduced-dose endoscopic tattoo.

**Figure 4.**
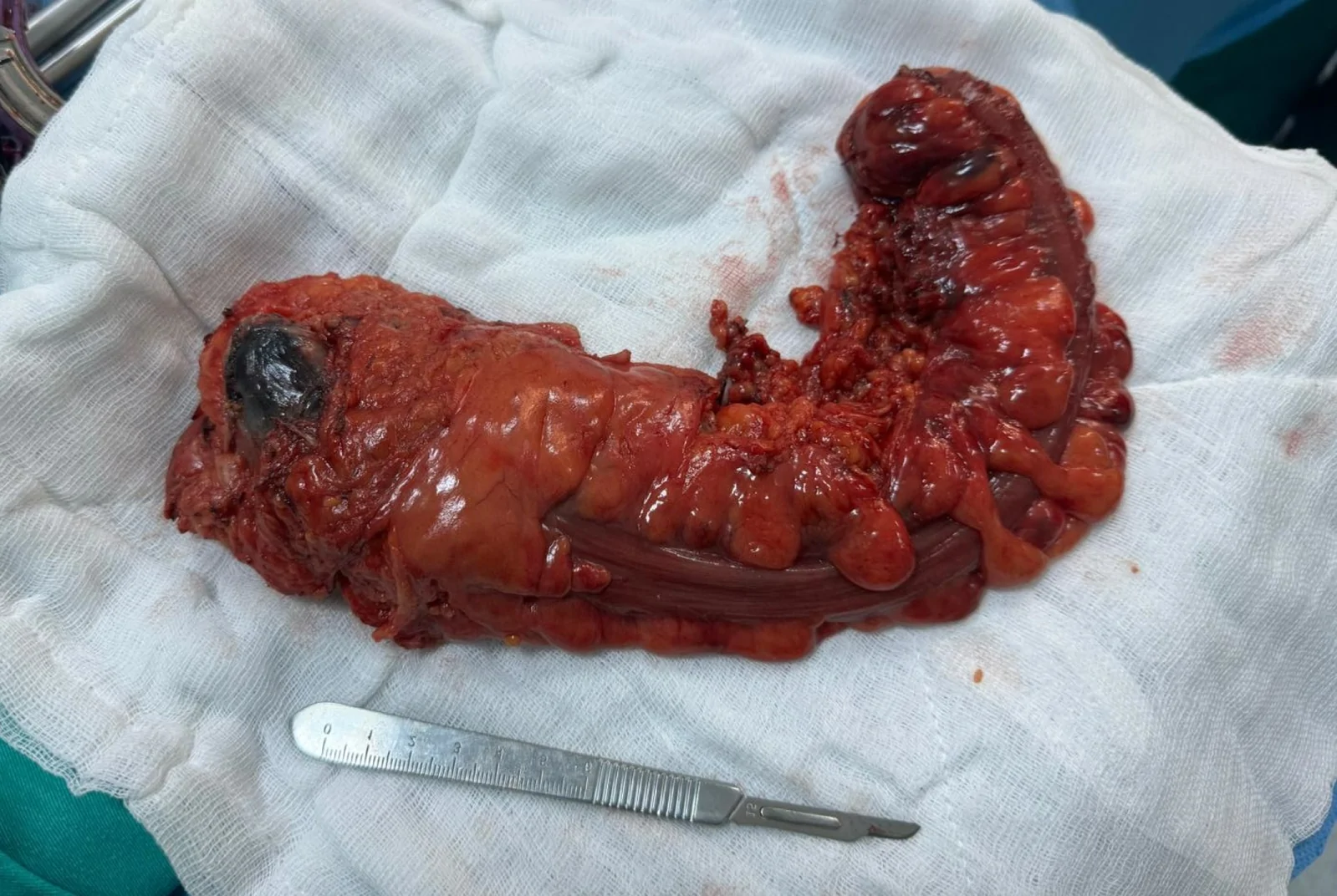
Macroscopic examination of the resected specimen with the tattooed area clearly visible on the colorectal wall and adjacent mesentery.

### 2.6. Ease of identification

The ease of tattoo identification was independently assessed and agreed upon by the operating surgeon and the first surgical assistant immediately upon entry into the abdominal cavity. Visibility was classified using a standardized binary scoring system:

- Easy identification: Defined as the immediate, effortless visualization of the carbon dye staining on the colonic/rectal serosa without requiring any additional surgical maneuvers or tissue dissection.
- Difficult/Failed identification: Defined as faint, dispersed, or completely absent visualization of the dye upon initial inspection, necessitating further intraoperative localization maneuvers.

For cases with difficult or failed initial identification, a standardized, stepwise rescue algorithm was strictly followed:

1. First, the suspected colon or rectum segment was carefully dissected laparoscopically to expose any deep or subserosal dye staining.
2. Second, if the dye remained unseen, a detailed laparoscopic inspection and instrumental palpation of the colonic wall were performed to identify the tumor mass or associated serosal puckering.
3. Third, if localization still could not be achieved, intraoperative colonoscopy was performed as the final definitive modality to precisely locate the tumor.

### 2.7. Statistical analysis

Descriptive statistics were calculated to summarize the demographic and clinical characteristics of the study sample. Continuous variables (such as age) were expressed as mean ± standard deviation (M±SD) or as median (minimum–maximum). Categorical variables were presented as frequency and percentage (%).

The normality of each numerical variable was assessed using the Kolmogorov-Smirnov test. Nonparametric continuous variables were presented as median (min–max). The relationship between 2 independent categorical variables was examined with the Chi-square test, and Fisher’s Exact Chi-square test was used when the assumptions for the Chi-square test were not met.

For comparisons among 3 groups, 1-way analysis of variance was used for parametric variables, while the Kruskal-Wallis test was applied for nonparametric variables. Homogeneity of variances was assessed using Levene’s test.

All statistical analyses were performed using the Statistical Package for the Social Sciences **−**22 statistical software package, and a *P* value of  < .05 was considered statistically significant.

Additionally, a multivariable logistic regression analysis was performed to identify independent predictors of failed or difficult intraoperative tattoo identification. The model adjusted for potential confounding factors, including BMI, tumor localization, T stage, neoadjuvant therapy, and tattooing dose groups. To ensure the absence of multicollinearity among the independent variables in the model, multicollinearity diagnostics were performed using Variance Inflation Factor values, with a VIF < 5.0 indicating acceptable collinearity. Missing BMI values were imputed using median imputation to maintain the statistical power of the model.

## 3. Results

A total of 116 patients were included in the study; 37 (31.9%) were female and 79 (68.1%) were male. The mean age of the patients was 63.8 (10.5) years. Regarding tumor localization in the colon, the most common sites were the rectosigmoid, sigmoid, or descending colon in 52 patients (44.8%); the rectum in 50 patients (43.1%); and synchronous metastases involving the lung, liver, or colon in 12 patients (10.3%) (Table 1).

**Table 1:** General characteristics of the patients.

Age (SD), yr	63.8 (10.5)
**Gender (total 116**)	
Female	37 (%31.9)
Male	79 (%68.1)
**Tumor localization**	
Rectosigmoid/sigmoid colon/descending colon	52 (44.8%)
Rectum	50 (43.1%)
Cecum/ascending colon/hepatic flexure	10 (8.6%)
Transverse colon/splenic flexure	2 (%1.7)
FAP/AFAP	2 (%1.7)
**Distant metastasis status**	
M0 (Nonmetastatic)	104 (89.7%)
M1 (Synchronous lung/liver/colon metastasis)	12 (10.3%)
**Type of surgery**	
Laparoscopic LAR/ Anterior Resection (with/without adjunctive procedures)*	88 (75.9%)
Laparoscopic Left Hemicolectomy (with/without adjunctive procedures)	15 (12.9%)
Laparoscopic Right/ Extended Right Hemicolectomy	11 (9.5%)
Laparoscopic Total Colectomy/ Proctocolectomy	2 (1.7%)
Ease of identification	103/116 (%88.7)

AFAP *=* Attenuated Familial Adenomatous Polyposis, FAP *=* Familial Adenomatous Polyposis, LAR *=* Laparoscopic Low Anterior Resection, SD *=* Standard Deviation.

* Includes cases with concurrent pelvic lymphadenectomy, gynecological resections (such as total abdominal hysterectomy and bilateral salpingo-oophorectomy), or liver/lung metastasectomy/ablation.

All patients underwent laparoscopic procedures. Based on the primary oncological resection, the most frequently performed procedures were laparoscopic low anterior resection or anterior resection (including cases with concurrent pelvic lymphadenectomy, gynecological resections, or liver/lung metastasectomy/ablation) in 88 patients (75.9%), laparoscopic left hemicolectomy in 15 patients (12.9%), and laparoscopic right or extended right hemicolectomy in 11 patients (9.5%) (Table 1). Patients with lower ease of identification often had advanced TNM and T stages (Table 2).

**Table 2:** Histopathological characteristics of the patients.

Variables	n (%)
Resected tumor diameter (cm)* (median)	4 (0.5–20)
Length of resected specimen (cm)	20 (6–90)
Malignancy rate	92/116 (%90.5)
TNM staging	
0†	24 (%20.7)
1	18 (%15.5)
2	36 (%31)
3	31 (%26.7)
4	7 (%6)
T stage	
0^†^	24 (%20.7)
1	6 (%5.1)
2	19 (%16.3)
3	48 (%41.3)
4	19 (%16.3)

* : *Largest tumor diameter.*

* *: median T: Primary tumor size or direct invasion, TNM staging: Staging of malignant tumors (T: size or direct invasion, N: lymph node involvement, M: metastasis), med: median, TNM Staging 0.*

* : *Carcinoma* in situ *or endoscopically unresectable polyps, T stage 0.*

† *: Carcinoma* in situ *or endoscopically unresectable polyps.*

Among the histopathological characteristics of the patients, the median resected tumor diameter was 4 cm (0.5–20), the median specimen length was 20 cm (6–90), and the malignancy rate was 90.5% (92/116). Histopathological examination showed that the most common TNM stages were stage 2 in 31%, stage 3 in 26.7%, stage 0 (carcinoma in situ or high-grade dysplasia) in 20.7%, and stage 1 in 15.5%. In addition, the most frequent T stage was T3 in 41.3%, followed by T0 or polyps not endoscopically resectable in 20.7%, T2 in 16.3%, and T4 in 16.3% (Table 2).

Statistical analysis among the groups revealed no significant differences in age, sex, BMI, American Society of Anesthesiologists score, tumor localization, operative time, median resected tumor diameter, malignancy rate, neoadjuvant therapy, TNM stage, median number of resected lymph nodes, or T stage (*P* > .05) (Table 3). Furthermore, there were no statistically significant differences among the 3 groups regarding ease of identification (*P* = .706). There were also no differences between the groups in terms of dye spillage into the peritoneum. In addition, no cases of peritonitis or allergic reactions related to peritoneal dissemination of the dye were observed (Table 3).

**Table 3:** Comparison of groups according to clinical and histopathological characteristics.

	Group 1 (≥1 cc) (n = 35)	Group 2 (1–0.5 cc) (n = 33)	Group 3 (<0.5 cc) (n = 48)	*p* value
Age (yr)	62 (9.560)	63 (11.338)	65 (10.439)	.465
Gender				.062
Female	9 (%25.7)	12 (%36.3)	16 (%33.4)	
Male	26 (%74.3)	21 (%63.7)	32 (%66.6)	
Body mass index (kg/m^2^)	27.638 ± 5.201	27.244 ± 3.096	25.65 ± 5.469	.237
ASA score	2.5 (2–3)	3 (2–3)	3 (2–3)	.146
Tumor localization				.908
Ease of identification	32 (%91.4)	30 (%90.9)	41 (%85.4)	.706
Operative time	270 (170–500)	250 (180–450)	265 (150–650)	.566
Tumor diameter*	4.32 (1.658)	4.95 (3.582)	3.58 (2.038)	.069
Malignancy rate	26 (%74.3)	29 (%87.9)	37 (%77.1)	.338
Neoadjuvant therapy	9 (%7.8)	6 (%5.2)	15 (%12.9)	.423
TNM stage	2 (0–3)	2 (0–4)	2 (0–4)	.219
Resected lymph nodes	12 (0–54)	14 (3–58)	13.5 (4–32)	.187
*T* stage	3 (0–4)	3 (0–4)	2 (0–4)	.290
Peritoneal spillage	5 (%14.3)	3 (%9.1)	2 (%4.2)	.267

* ASA *=* American Society of Anesthesiologists*, T =* Primary tumor size or direct invasion, TNM staging *=* Staging of malignant tumors (*T*: size or direct invasion, N: lymph node involvement, M: metastasis).

Furthermore, to address potential confounding related to the inclusion of nonmalignant cases (such as non-resectable polyps or benign tumors, n = 24), a sensitivity analysis was performed by restricting the cohort strictly to patients with histopathologically proven invasive CRC (n = 92). In this malignant-only subgroup, the rate of easy intraoperative tattoo identification was 92.3% (24/26) in Group 1, 89.7% (26/29) in Group 2, and 81.1% (30/37) in Group 3. Similar to the primary analysis, no statistically significant difference in ease of identification was observed among the 3 dose groups within the invasive cancer cohort (*P* = .373).

To address potential confounding factors for failed or difficult intraoperative tattoo identification, a multivariable logistic regression model was constructed. The multivariable analysis demonstrated that none of the clinical parameters, including tattooing dose groups (Group 2: OR = 1.27, *P* = .789; Group 3: OR = 1.82, *P* = .438), tumor localization, neoadjuvant therapy, T stage, or BMI, were independent risk factors for failed or difficult identification (all *P* > .05) (Table 4).

**Table 4:** Multivariable logistic regression analysis of factors associated with failed/difficult intraoperative tattoo identification.

Variable	Odds ratio (OR)	95% Confidence interval (CI)	*p*-value
**Dose group**			
- Group 1 (≥1.0 cc)	*Reference*	–	–
- Group 2 (0.5 – < 1.0 cc)	1.27	0.22–7.36	.789
- Group 3 (<0.5 cc)	1.82	0.40–8.35	.438
**Tumor Localization**			
- Left colon *(including transverse*)	*Reference*	–	–
- Rectum	0.46	0.09–2.38	.356
- Right Colon	0.90	0.09–9.12	.929
- Other *(FAP/AFAP*)	5.65	0.35–91.62	.223
Neoadjuvant therapy *(Yes vs No*)	2.12	0.41–11.00	.373
*T* Stage	1.08	0.67–1.72	.760
BMI (kg/m^2^)*	0.96	0.83–1.12	.626

* *Missing BMI values* (n* = *29*) were imputed with the cohort median* (26.1 kg/m^2^*) to preserve statistical power. Note: Time interval (tattoo-to-surgery) was excluded from the multivariable model as it was standardized to 1 to 3 days for all patients* (0 *variance).*

## 4. Discussion

In our study, which included 116 patients who underwent endoscopic tattooing with different dye doses using a standard protocol followed by laparoscopic surgery, easy identification was achieved in 88.7% of patients without any adverse events. When we compared the ease of identification rates among the groups, it was 91.4% in Group 1 (≥1.0 cc), 90.9% in Group 2 (0.5–<1.0 cc), and 85.4% in Group 3 (<0.5 cc), with no statistically significant differences. In our study, the overall ease of identification rate was 88.7%, while the 85.4% rate in Group 3 was not only consistent with the literature but also frequently exceeded previously reported outcomes.^[1,3,4,6,10]^

We believe that the main distinguishing factor here is that both the endoscopic tattooing and the laparoscopic surgery were performed by the same surgeon, which increased the visibility of the dye during the operation regardless of the amount used. Having both procedures performed by the same surgeon allows for better familiarity with the patient’s imaging studies, such as computed tomography or magnetic resonance imaging, and increases the likelihood of immediate intraoperative identification. Our finding that tattooing and laparoscopic surgery were performed by the same surgeon represents a significant methodological strength, ensuring high internal standardization and minimizing operator-dependent bias. However, this center-specific factor also limits the generalizability of our results to institutions where preoperative marking and oncological resections are routinely conducted by different clinical teams or specialists.

In the literature, there are discussions regarding the substance, dose, technique, site, and timing of endoscopic tattooing in laparoscopic CRC surgery. In the study by Arteaga et al., India ink was used preoperatively in doses of 0.2 to 0.5 mL in the days before surgery. Tattooing was performed at a single site depending on the lesion, and the procedure was carried out by multiple different endoscopists. In this study, the group with dye tattooing achieved correct surgical procedures in all patients due to improved visual identification.^[3]^ In the retrospective study by Milsom et al., which included 58 patients with colorectal polyps and tumors, clips and dye (India ink) were used, tattooing was performed before surgical treatment, and ease of identification was achieved in 53.4% of patients, while additional procedures were required to determine tumor localization in the others.^[4]^ In another study by Kirchoff et al, which included 27 patients who underwent anterior resection for sigmoid colon and rectal tumors, the distal surgical margin was tattooed with dye, and the ease of identification rate was reported to be above 90%.^[6]^ In the study by Conaghan et al on laparoscopic colorectal surgery, although the ease of identification rates were similar between groups with and without dye tattooing, complication rates were statistically lower in the group with dye tattooing.^[10]^ The most important conclusion from these studies is that the use of dye for tattooing improves ease of identification and reduces complication rates.

On the other hand, the limited number of studies comparing different tattooing doses has led to the need for new research. Therefore, the larger number of patients in our study, the fact that all tattooing procedures were performed by the same endoscopist, and the evaluation of outcomes with different doses introduce an important innovation regarding ease of identification in laparoscopic colorectal surgery.

In our study, it was also shown that in patients with CRC who received total neoadjuvant therapy or chemoradiotherapy, the presence of scar tissue or residual tumor tissue did not create a statistically significant difference in ease of identification with different tattooing doses, and that reduced-dose dye tattooing could be safely used in patients receiving neoadjuvant therapy.^[11]^ Although the number of patients who underwent neoadjuvant chemoradiotherapy was higher in the reduced-dose group (Group 3, <0.5 cc), there was no statistically significant difference in the ease of identification rate. Therefore, in polyps resected endoscopically and considered potentially malignant, reduced-dose endoscopic tattooing performed 1 to 2 cm distal to the polypectomy site may reduce the need for unnecessary preoperative colonoscopies.^[12]^

One of the important aspects of our study is the homogeneous distribution of groups that received different amounts of endoscopic dye and its design as a comparative study. One of the main factors reducing the ease of identification during surgery is a high BMI. In patients with high BMI, an increase in intra-abdominal adipose tissue was observed, which was associated with hypertrophy of the colonic mesentery. However, since the mean BMI was similar across all groups in our study, this factor was not statistically significant.

After endoscopic tattooing, especially when high doses are used, spillage of the dye into the peritoneum, inflammatory reactions, peritonitis, bowel obstruction, microhemorrhage, and thrombosis may occur.^[8,13–16]^ Although India ink is inexpensive, it has been reported to be associated with allergic reactions. In the review by Nizam et al, an inflammatory reaction rate of 0.22% was reported due to tattooing with India ink.^[14]^ In addition, Falco et al reported a case of focal peritonitis and ileus associated with India ink.^[15]^ In the review by Pontone et al on tattooing/clip marking with indocyanine green, the method was reported to be safe in terms of complications. Indocyanine green marking is also frequently used for lymph node mapping and resection.^[16]^ In the study by Ho et al involving indocyanine green tattooing, a peritoneal spillage rate of 14.2% was reported.^[17]^

While our study demonstrates the efficacy of carbon-based liquid tattooing, alternative localization strategies have gained prominence in minimally invasive surgery. These include advanced near-infrared fluorescent imaging using indocyanine green and magnetic ring markers, which provide high precision without the risk of peritoneal staining seen in traditional dyes, but require specialized optical equipment and higher procurement costs. Additionally, while intraoperative endoscopy offers active localization and margin verification, it increases operative complexity. This makes carbon-based tattooing with refined doses a highly accessible, cost-effective, and safe alternative in standard laparoscopic settings.^[17–19]^

In our study, we used Spot® as the dye, and no major complications other than peritoneal spillage were observed. The main reasons for this were that the procedure was performed 1 to 3 days before surgery, that we diluted the dye by administering 1 to 3 cc of saline before injection, and that injections were always made at an angle of 10 to 15 degrees to the mucosa. With this approach, extravasation outside the colonic or rectal wall is very rare. The peritoneal spillage rate of tattooing in our study was 8.6%, which was more favorable compared with the literature. Regarding the cost analysis, while Spot® is supplied in single-use vials, the cost advantage of reduced-dose application is particularly realized in high-volume reference centers. In our institutional workflow, scheduling consecutive procedures on the same clinical day allows for the efficient and sterile portioning of a single vial for multiple scheduled patients, thereby significantly minimizing vial wastage and reducing overall healthcare expenditure.

Our study has some limitations. In particular, its retrospective nature may have influenced the distribution of data. In addition, the small number of patients in whom marking was performed for cecal and ascending colon tumors because the anatomical localization was clearly evaluated represents another limitation. Third, although our study included a minor subset of nonmalignant tumors/polyps alongside invasive CRC, our subgroup sensitivity analysis demonstrated that this case-mix did not confound our primary findings regarding the efficacy of low-dose tattooing.

## 5. Conclusion

In laparoscopic CRC or tumor surgery, reduced-dose tattooing is as effective as high-dose in ease of identification. Reduced doses provide cost, labor, and safety advantages by lowering peritoneal spillage.

## Author contributions

**Conceptualization:** Ali İmran Küçük, Mehmet Aziret.

**Methodology:** Ali İmran Küçük, Onur Sakalli, Mehmet Aziret.

**Data curation:** Bülent Toksöz.

**Investigation:** Bülent Toksöz.

**Resources:** Bülent Toksöz, Mehmet Aziret.

**Project administration:** Mehmet Aziret.

**Supervision:** Mehmet Aziret.

**Writing – original draft:** Ali İmran Küçük, Onur Sakalli.

**Writing – review & editing:** Ali İmran Küçük, Onur Sakalli.
